# Diabetes-free survival among living kidney donors and non-donors with obesity: A longitudinal cohort study

**DOI:** 10.1371/journal.pone.0276882

**Published:** 2022-11-18

**Authors:** A. Cozette Killian, Rhiannon D. Reed, M. Chandler McLeod, Paul A. MacLennan, Vineeta Kumar, Sydney E. Pittman, Andrew G. Maynor, Luke A. Stanford, Gavin A. Baker, Carrie A. Schinstock, John R. Silkensen, Garrett R. Roll, Dorry L. Segev, Babak J. Orandi, Cora E. Lewis, Jayme E. Locke

**Affiliations:** 1 Comprehensive Transplant Institute, University of Alabama at Birmingham, Birmingham, AL, United States of America; 2 Division of Nephrology and Hypertension, Mayo Clinic, Rochester, Minnesota, United States of America; 3 Department of Medicine, Hennepin County Medical Center, Minneapolis, Minnesota, United States of America; 4 Division of Transplant, Department of Surgery, University of California San Francisco, San Francisco, CA, United States of America; 5 Department of Surgery, New York University Grossman School of Medicine, New York, New York, United States of America; 6 Department of Epidemiology, School of Public Health, University of Alabama at Birmingham, Birmingham, AL, United States of America; Imperial College Healthcare NHS Trust, UNITED KINGDOM

## Abstract

**Background:**

Approval of living kidney donors (LKD) with end-stage kidney disease (ESKD) risk factors, such as obesity, has increased. While lifetime ESKD development data are lacking, the study of intermediate outcomes such as diabetes is critical for LKD safety. Donation-attributable diabetes risk among persons with obesity remains unknown. The purpose of this study was to evaluate 10-year diabetes-free survival among LKDs and non-donors with obesity.

**Methods:**

This longitudinal cohort study identified adult, LKDs (1976–2020) from 42 US transplant centers and non-donors from the Coronary Artery Risk Development in Young Adults (1985–1986) and the Atherosclerosis Risk in Communities (1987–1989) studies with body mass index ≥30 kg/m^2^. LKDs were matched to non-donors on baseline characteristics (age, sex, race, body mass index, systolic and diastolic blood pressure) plus diabetes-specific risk factors (family history of diabetes, impaired fasting glucose, smoking history). Accelerated failure time models were utilized to evaluate 10-year diabetes-free survival.

**Findings:**

Among 3464 participants, 1119 (32%) were LKDs and 2345 (68%) were non-donors. After matching on baseline characteristics plus diabetes-specific risk factors, 4% (7/165) LKDs and 9% (15/165) non-donors developed diabetes (median follow-up time 8.5 (IQR: 5.6–10.0) and 9.1 (IQR: 5.9–10.0) years, respectively). While not significant, LKDs were estimated to live diabetes-free 2 times longer than non-donors (estimate 1.91; 95% CI: 0.79–4.64, p = 0.15).

**Conclusions:**

LKDs with obesity trended toward living longer diabetes-free than non-donors with obesity, suggesting within the decade following donation there was no increased diabetes risk among LKDs. Further work is needed to evaluate donation-attributable diabetes risk long-term.

## Introduction

By 2030, the prevalence of end-stage kidney disease (ESKD) in the United States is estimated to exceed 970,000 [1]. Compared to dialysis, kidney transplantation is the best treatment option for ESKD, affording improved survival and quality of life at reduced healthcare costs [2–4]. However, a tremendous supply/demand mismatch remains for kidney transplantation [5]. With fewer than 18,000 deceased donor kidney transplants performed annually, the additional ~7,000 living donor kidney transplants (LDKT) are a critical to the kidney supply, yet insufficient to meet demand [6]. Importantly, greater LDKT utilization is essential to address the goals of the 2019 Advancing American Kidney Health executive order, which calls for doubling of kidneys available for transplant by 2030 [7, 8].

Comorbid diseases that fuel the increasing ESKD prevalence also impact the living kidney donor (LKD) supply. More than 40% of adults suffer from obesity [9], which increases their risk of diabetes [10, 11], the primary cause of ESKD in the United States [12]. Efforts to increase LDKT have prompted the acceptance of complex LKD, defined by Reese et al. as LKDs with ESKD risk factors, such as obesity [13]. While LKDs with obesity have become more prevalent–nearly 25% of LKDs nationally have obesity, the long-term safety of donation in this population is unknown [14–16]. Our understanding of the impact of kidney donation on lifetime ESKD development–the guiding principle of donor selection–remains limited as most studies report mean follow-up of 20 years or less [17–19]. Given the concern for kidney disease development or progression in the setting of diabetes and uninephrectomy [20–22], the need to evaluate intermediate outcomes of ESKD, such as diabetes development among LKDs with obesity, is critical to guide donation practices and prioritize LKD safety.

Similar to findings in the general population [11], studies suggest LKDs with obesity have greater risk of diabetes development compared to LKDs who are not obese [18, 21, 23, 24]. While these comparisons of LKDs confirm obesity as a diabetes risk factor, donation-associated diabetes risk among obese individuals requires comparison of LKDs and healthy non-donors, and remains unknown [19]. The 2017 Kidney Disease: Improving Global Outcome practice guidelines for living donation outline the rigorous testing required for LDKs, and emphasize the need for greater understanding of donation-attributable risk as part of its framework for LKD selection [19]. While data regarding the metabolic impact of donor nephrectomy are sparse, it is well-established that kidneys maintain a vital role in both gluconeogenesis and insulin clearance [25–27]. Thus, it is plausible that LKDs experience altered insulin and glucose metabolism post-donation.

Consequently, an understanding of donation-attributable diabetes risk among individuals with obesity would inform LKD selection, facilitate informed consent, appropriate follow-up, and long-term care for the growing population of LKDs with obesity. Utilizing a national cohort of LKDs with obesity, we match LKDs to non-donors from two well-established longitudinal studies (Coronary Artery Risk Development in Young Adults (CARDIA)) and the Atherosclerosis Risk in Communities (ARIC)) to evaluate 10-year diabetes-free survival. Despite the potential for metabolic alterations among LKDs, we hypothesized there would be no significant difference in diabetes development between LKDs and non-donors with obesity given the rigorous pre-donation evaluation.

## Methods

### Data sources and study population

#### Donors with obesity

In this longitudinal cohort study, donors were identified from two NIH-funded studies (1R01DK113980, Locke; 1R01096008, Segev). Donors underwent living kidney donation between September 1976 and May 2020 and were recruited via two possible mechanisms. If the donor’s transplant center agreed to participate, the center mailed a recruitment letter. Other donors were recruited via a letter sent by research personnel at the University of Alabama at Birmingham after obtaining a waiver for authorization and consent to receive donor contact information from the Organ Procurement and Transplantation Network (OPTN)/Scientific Registry of Transplant Recipients (SRTR). Of the 6,118 LKDs who were successfully contacted, 1,119 were eligible for inclusion in our analyses (S1 Fig).

This study used data from the SRTR. The SRTR data system includes data on all donors, wait-listed candidates, and transplant recipients in the US, submitted by the members of the OPTN. The Health Resources and Services Administration (HRSA), U.S. Department of Health and Human Services provides oversight to the activities of the OPTN and SRTR contractors. Study data were collected and managed using REDCap electronic data capture tools hosted at the University of Alabama at Birmingham (UAB) [28]. This study adhered to the Declaration of Helsinki, was approved by the UAB Institutional Review Board, and all participants provided informed consent (IRB-300000039; IRB-131003001).

#### Non-donors with obesity

Non-donors were identified from the CARDIA and ARIC studies, which have been previously described [29, 30]. Briefly, CARDIA enrolled participants aged 18–30 years in 1985–6, while ARIC enrolled participants aged 45–65 years in 1987–89. To identify non-donors aged 30–45 years and older than 65 years, CARDIA and ARIC participants contributed multiple non-donor records based on follow-up exams. Thus, CARDIA participants contributed up to 5 records including baseline exam and 10-, 15-, 20-, and 25-year follow-up exams, while ARIC participants contributed up to 3 records including baseline exam and 3- and 9-year follow-up exams. All records were assessed for LKD eligibility, as previously described (S2, S3 Figs) [31].

### Outcomes and exposures

#### Inclusion criteria and exposure definitions

Donor and non-donor records were included if baseline body mass index (BMI) was ≥30 kg/m^2^. Donor baseline BMI was defined as BMI at donation. Donor pre-operative characteristics were obtained from medical record abstraction and the SRTR living donor file. Additional data collected among donors and frequency of medical follow-up is included in the S1 File and S1–S3 Tables. Non-donor baseline BMI was utilized from the relevant eligible exam as described above. Impaired fasting glucose was defined as a baseline fasting blood glucose 100–125 mg/dL or baseline A1c 5.6–6.4%. Donors and non-donors were excluded for baseline fasting blood glucose ≥126 mg/dL, baseline A1c ≥6.5%, or prior diagnosis of diabetes. Donors with only SRTR data were excluded from primary analyses. All records were followed from donation (donors) or eligible exam (non-donors) until the earliest of diabetes development, latest follow-up exam, or 10 years.

#### New-onset diabetes outcome

New-onset diabetes was defined as ≥1 of the following: self-reported diabetes diagnosis or medication indicated for diabetes (including Metformin and insulin), two or more consecutive random blood glucose measurements ≥200 mg/dL, a single fasting blood glucose ≥126 mg/dL, or a single hemoglobin A1C ≥6.5%. For donors only, any new diabetes diagnosis in their medical record or SRTR living donor follow-up file was also utilized (S4, S5 Tables). Individuals with new-onset diabetes within 10 years were interval-censored using the last observation without and earliest observation with diabetes, respectively. The latter was defined using 1) date of elevated glucose measurement, 2) date of first evidence of diabetes diagnosis in the medical record, 3) self-reported year or age of diabetes diagnosis, or 4) survey date if patient confirmed diabetes diagnosis but self-reported year was unavailable.

### Statistical analysis

#### Matching

We performed matching without replacement to balance baseline characteristics between donors (n = 1119) and non-donor records (n = 3778). A 1:1 donor to non-donor ratio with exact matching on categorical covariates (race, sex, family history of diabetes, impaired fasting glucose, smoking history) and caliper matching on continuous covariates (age, systolic blood pressure (SBP), diastolic blood pressure (DBP), BMI) was used. Age was matched within 3 years, BMI within 2 kg/m^2^, and SBP and DBP within 5 mmHg. Because of missing data for family history of diabetes (donors: 30% (334/1119), non-donors: 19% (706/3778)), impaired fasting glucose (donors: 56% (629/1119), non-donors: 5% (174/3778)), and smoking history (donors: 8% (90/1119), non-donors: (<1%) 17/3778) two matched cohorts were formed for analyses. The first cohort was matched on baseline characteristics (age, sex, race, BMI, SBP, DBP) only. The distribution of non-donor records matched to donors on baseline characteristics is included in S6 Table. The second cohort was matched on baseline characteristics plus diabetes-specific risk factors (family history of diabetes, impaired fasting glucose, smoking history). Match balance was assessed by standardized mean differences using a threshold of 0.1. To ensure independence among records and maximize 10-year follow up, each matched cohort was limited to unique persons using the youngest non-donor record and corresponding matched donor.

#### Descriptive analyses

Baseline characteristics were evaluated for unique donors and non-donors prior to and after matching. Bivariate comparisons were performed by donor status, utilizing t-tests for continuous variables and chi-squared tests for categorical variables.

#### Non-parametric survival methods

For both matched cohorts, non-parametric survival methods were used to compare 10-year diabetes-free survival among donors and non-donors. Estimates of the survival distribution were performed with accommodation for interval censoring, and the log-rank test was used for comparisons between donor/non-donor survival curves [32].

#### Accelerated failure time models

To quantify differences in 10-year diabetes-free survival between donors and non-donors, both matched cohorts were analyzed using Weibull accelerated failure time (AFT) models, which can account for interval censoring [33]. First, 10-year diabetes-free survival was modeled as a function of donor status using the cohort matched on baseline characteristics only. Second, using this same cohort, 10-year diabetes-free survival was modeled as a function of donor status while adjusting for family history of diabetes, impaired fasting glucose, and smoking history. If a donor or non-donor was missing data for any of these covariates, the matched pair was dropped from the model to maintain balance. Third, 10-year diabetes-free survival was modeled as a function of donor status in the cohort matched on baseline characteristics plus diabetes-specific risk factors.

### Sensitivity analyses

Descriptions of all sensitivity analyses are included in the S1 File. In all additional analyses, donors demonstrated lower risk of 10-year diabetes development or longer diabetes-free survival compared to non-donors (S7–S12 Tables; S4 Fig).

Analyses utilized SAS 9.4 (Cary, NC) and R version 4.0.2 (R Core Team, 2020; S1 File). All statistical tests were considered significant at p<0.05.

## Results

### Overall cohort baseline characteristics

This study included 3464 unique individuals from four studies, of which 1119 (32%) were LKDs and 2345 (68%) were non-donors (Table 1). Donors and non-donors were of similar baseline age (45.8 (standard deviation (SD) 11.0) vs. 44.8 (SD 11.8) years, p = 0.02). Both groups had similar proportions of females (62% vs. 60%, p = 0.21), though donors had larger proportions of White individuals (83% vs. 59%; p < .001). Donors and non-donors had similar baseline BMI (33.1 (SD 2.7) vs. 33.2 (SD 3.3) kg/m^2^, p = 0.34), but donors demonstrated higher baseline blood pressures (SBP: 124.4 (SD 12.6) vs. 114.4 (SD 10.6), p < .001); DBP: 75.6 (SD 8.9) vs. 71.8 (SD 7.8), p < .001). Among participants with cardiometabolic data, donors had significantly higher high-density lipoproteins (HDL) (53.3 (SD 16.3) vs. 47.3 (SD 13.6), p < .001), but similar triglycerides (127.2 (SD 66.1) vs. 122.9 (SD 84.4), p = 0.33). Among participants with data for diabetes-specific risk factors, family history of diabetes was more prevalent among donors (44% vs. 33%, p < .001), though impaired fasting glucose was similar among donors and non-donors (33% vs. 36%, p = 0.14). Smoking history was less prevalent among donors (30% vs. 48%, p < .001). Other baseline comparisons of donor to non-donor characteristics were statistically significant due to sample sizes, though differences were small and not clinically pertinent.

**Table 1:** Demographics and baseline characteristics of all unique living kidney donors (1R01DK113980 and 1R01096008) and non-donors (CARDIA and ARIC).

	Total	Donor	Non-donor	p-value
	(N = 3464)	(N = 1119)	(N = 2345)	
	N (%)	N (%)	N (%)	
Age (years), mean (SD)	45.1 (11.5)	45.8 (11.0)	44.8 (11.8)	0.02
Female Sex	2106 (60.8%)	697 (62.3%)	1409 (60.1%)	0.21
Race				< 0.001
Non-White	1151 (33.2%)	192 (17.2%)	959 (40.9%)	
White	2313 (66.8%)	927 (82.8%)	1386 (59.1%)	
BMI (kg/m^2^), mean (SD)	33.2 (3.1)	33.1 (2.7)	33.2 (3.3)	0.34
WHO class				< 0.001
Class I (30–34.9)	2754 (79.5%)	898 (80.3%)	1856 (79.1%)	
Class II (35–39.9)	552 (15.9%)	195 (17.4%)	357 (15.2%)	
Class III (40+)	158 (4.6%)	26 (2.3%)	132 (5.6%)	
SBP (mmHg), mean (SD)	117.6 (12.2)	124.4 (12.6)	114.4 (10.6)	< 0.001
DBP (mmHg), mean (SD)	73.0 (8.4)	75.6 (8.9)	71.8 (7.8)	< 0.001
Serum Creatinine, mean (SD)^a^	0.78 (0.17)	0.87 (0.19)	0.75 (0.15)	< 0.001
eGFR, mean (SD)^b^	105.4 (24.5)	93.1 (17.4)	111.2 (25.3)	< 0.001
HDL^c^	48.2 (14.2)	53.3 (16.3)	47.3 (13.6)	< 0.001
Triglycerides^d^	123.5 (81.9)	127.2 (66.1)	122.9 (84.4)	0.33
History of high cholesterol				0.001
No	1623 (86.4%)	631 (83.2%)	992 (88.5%)	
Yes	256 (13.6%)	127 (16.8%)	129 (11.5%)	
Missing	1585	361	1224	
Fasting blood glucose^e^	95.8 (11.4)	93.3 (12.9)	96.3 (11.0)	< 0.001
Impaired fasting glucose				0.14
No	1759 (64.5%)	330 (67.3%)	1429 (63.9%)	
Yes	969 (35.5%)	160 (32.7%)	809 (36.1%)	
Missing	736	629	107	
Family history of diabetes				< 0.001
No	1763 (63.6%)	438 (55.8%)	1325 (66.7%)	
Yes	1009 (36.4%)	347 (44.2%)	662 (33.3%)	
Missing	692	334	358	
Ever smoker				< 0.001
No	1947 (57.9%)	723 (70.3%)	1224 (52.4%)	
Yes	1418 (42.1%)	306 (29.7%)	1112 (47.6%)	
Missing	99	90	9	
Family history of hypertension				< 0.001
No	1302 (44.5%)	431 (54.6%)	871 (40.7%)	
Yes	1627 (55.5%)	358 (45.4%)	1269 (59.3%)	
Missing	535	330	205	

Abbreviations: SD = standard deviation; BMI = Body Mass Index; WHO = World Health Organization; SBP = Systolic blood pressure; DBP = Diastolic blood pressure; eGFR = estimated glomerular filtration rate; HDL = high density lipoproteins

Impaired fasting glucose: Baseline FBG 100–125 or A1c 5.6–6.4

^a^missing for 0.2% donors and 0% of non-donors

^b^missing for 0.2% donors and 0% of non-donors

^c^missing for 66% of donors and 0.8% of non-donors

^d^missing for 63% of donors and 0.7% of non-donors

^e^missing for 55% of donors and 4% of non-donors

#### Comparison of donors to matched non-donors

In the cohort matched on baseline characteristics only, donors and non-donors were well-balanced on matched characteristics, including age, sex, race, BMI, SBP and DBP (Table 2). Family history of diabetes was significantly more prevalent among donors (43% vs. 33%, p < .001), while impaired fasting glucose (31% vs. 40%, p = 0.01) and smoking history (29% vs. 46%, p < .001) were significantly less prevalent among donors. Donors had higher HDL (53.8 (SD 15.3) vs. 47.5 (SD 14.0), p < .001), but similar triglycerides (125.3 (SD 65.7) vs. 130.8 (SD 77.5), p = 0.31).

**Table 2:** Demographics and characteristics of unique living kidney donors (1R01DK113980 and 1R01096008) and non-donors (CARDIA and ARIC) matched on baseline characteristics*.

	Total	Donor	Non-donor	p-value
	(N = 1376)	(N = 688)	(N = 688)	
	N (%)	N (%)	N (%)	
Age, (years), mean (SD)	46.9 (10.2)	46.8 (10.3)	47.0 (10.2)	0.72
Female Sex	866 (62.9%)	433 (62.9%)	433 (62.9%)	1.00
Race				1.00
Non-White	276 (20.1%)	138 (20.1%)	138 (20.1%)	
White	1100 (79.9%)	550 (79.9%)	550 (79.9%)	
BMI (kg/m^2^), mean (SD)	32.7 (2.3)	32.7 (2.2)	32.7 (2.3)	0.72
WHO class				0.97
Class I (30–34.9)	1149 (83.5%)	575 (83.6%)	574 (83.4%)	
Class II (35–39.9)	208 (15.1%)	104 (15.1%)	104 (15.1%)	
Class III (40+)	19 (1.4%)	9 (1.3%)	10 (1.5%)	
SBP (mmHg), mean (SD)	120.0 (10.0)	120.4 (10.2)	119.6 (9.9)	0.13
DBP (mmHg), mean (SD)	74.1 (7.2)	74.2 (7.3)	74.1 (7.1)	0.80
Serum Creatinine, mean (SD)^a^	0.79 (0.18)	0.86 (0.18)	0.72 (0.15)	< 0.001
eGFR, mean (SD)^b^	101.2 (32.0)	93.3 (17.2)	109.0 (40.4)	< 0.001
HDL^c^	49.2 (14.6)	53.8 (15.3)	47.5 (14.0)	< 0.001
Triglycerides^d^	129.3 (74.5)	125.3 (65.7)	130.8 (77.5)	0.31
History of high cholesterol				0.38
No	672 (83.5%)	402 (82.5%)	270 (84.9%)	
Yes	133 (16.5%)	85 (17.5%)	48 (15.1%)	
Missing	571	201	370	
Fasting blood glucose^e^	96.1 (11.4)	93.3 (12.4)	97.5 (10.6)	< 0.001
Impaired fasting glucose				0.01
No	606 (62.9%)	208 (68.6%)	398 (60.2%)	
Yes	358 (37.1%)	95 (31.4%)	263 (39.8%)	
Missing	412	385	27	
Family history of diabetes				0.001
No	653 (62.3%)	286 (57.3%)	367 (66.8%)	
Yes	395 (37.7%)	213 (42.7%)	182 (33.2%)	
Missing	328	189	139	
Ever smoker				< 0.001
No	825 (62.4%)	452 (71.3%)	373 (54.2%)	
Yes	497 (37.6%)	182 (28.7%)	315 (45.8%)	
Missing	54	54	0	
Family history of hypertension				< 0.001
No	541 (48.5%)	283 (56.7%)	258 (41.9%)	
Yes	574 (51.5%)	216 (43.3%)	358 (58.1%)	
Missing	261	189	72	

*Donors matched to non-donors on sex, race, age (+/-3 years), BMI (+/-2 kg/m^2^), Baseline SBP (+/-5 mmHg), Baseline DBP (+/-5 mmHg)

Abbreviations: SD = standard deviation; BMI = Body Mass Index; WHO = World Health Organization; SBP = Systolic blood pressure; DBP = Diastolic blood pressure; eGFR = estimated glomerular filtration rate; HDL = high density lipoproteins

Impaired fasting glucose: Baseline FBG 100–125 or A1c 5.6–6.4

^a^missing for 0.1% donors and 0% of non-donors

^b^missing for 0.1% donors and 0% of non-donors

^c^missing for 64% of donors and 0.7% of non-donors

^d^missing for 63% of donors and 0.5% of non-donors

^e^missing for 55% of donors and 4% of non-donors

In the cohort matched on baseline characteristics plus diabetes-specific risk factors, there were no significant differences between donors and non-donors in any matched characteristic including age, sex, race, BMI, SBP and DBP, family history of diabetes, impaired fasting glucose, or smoking history (Table 3). Donors had higher HDL (53.3 (SD 16.3) vs. 48.7 (SD 15.4), p = 0.02), but similar triglycerides (130.9 (SD 73.4) vs. 125.7 (SD 59.3), p = 0.52).

**Table 3:** Demographics and characteristics of unique living kidney donors (1R01DK113980 and 1R01096008) and non-donors (CARDIA and ARIC) matched on baseline characteristics plus diabetes-specific risk factors*.

	Total	Donor	Non-donor	p value
	(N = 330)	(N = 165)	(N = 165)	
	N (%)	N (%)	N (%)	
Age (years), mean (SD)	491 (8.3)	48.9 (8.3)	49.2 (8.2)	0.73
Female Sex	196 (59.4%)	98 (59.4%)	98 (59.4%)	1.00
Race				1.00
Non-White	66 (20.0%)	33 (20.0%)	33 (20.0%)	
White	264 (80.0%)	132 (80.0%)	132 (80.0%)	
BMI (kg/m^2^), mean (SD)	32.9 (2.1)	32.7 (2.1)	33.0 (2.2)	0.38
WHO class				0.67
Class I (30–34.9)	274 (83.0%)	140 (84.8%)	134 (81.2%)	
Class II (35–39.9)	54 (16.4%)	24 (14.5%)	30 (18.2%)	
Class III (40+)	2 (0.6%)	1 (0.6%)	1 (0.6%)	
SBP (mmHg), mean (SD)	120.8 (8.8)	120.9 (8.9)	120.6 (8.7)	0.74
DBP (mmHg), mean (SD)	75.1 (6.1)	75.0 (6.2)	75.2 (5.9)	0.78
Serum Creatinine, mean (SD)	0.80 (0.18)	0.87 (0.18)	0.73 (0.14)	< 0.001
eGFR, mean (SD)	99.2 (16.0)	92.1 (17.6)	106.2 (10.1)	< 0.001
HDL^a^	50.5 (15.9)	53.3 (16.3)	48.7 (15.4)	0.02
Triglycerides^b^	127.8 (65.2)	130.9 (73.4)	125.7 (59.3)	0.52
History of high cholesterol				0.77
No	172 (78.2%)	129 (78.7%)	43 (76.8%)	
Yes	48 (21.8%)	35 (21.3%)	13 (23.2%)	
Missing	110	1	109	
Fasting blood glucose^c^	95.8 (11.3)	93.3 (11.9)	98.0 (10.2)	< 0.001
Impaired fasting glucose				1.00
No	212 (64.2%)	106 (64.2%)	106 (64.2%)	
Yes	118 (35.8%)	59 (35.8%)	59 (35.8%)	
Family history of diabetes				1.00
No	200 (60.6%)	100 (60.6%)	100 (60.6%)	
Yes	130 (39.4%)	65 (39.4%)	65 (39.4%)	
Ever smoker				1.00
No	216 (65.5%)	108 (65.5%)	108 (65.5%)	
Yes	114 (34.5%)	57 (34.5%)	57 (34.5%)	
Family history of hypertension				0.13
No	174 (53.5%)	94 (57.7%)	80 (49.4%)	
Yes	151 (46.5%)	69 (42.3%)	82 (50.6%)	
Missing	5	2	3	

*Donors matched to non-donors on sex, race, family history of diabetes, impaired fasting glucose, smoking history, age (+/-3 years), BMI (+/-2 kg/m^2^), Baseline SBP (+/-5 mmHg), Baseline DBP (+/-5 mmHg)

Abbreviations: SD = standard deviation; BMI = Body Mass Index; WHO = World Health Organization; SBP = Systolic blood pressure; DBP = Diastolic blood pressure; eGFR = estimated glomerular filtration rate; HDL = high density lipoproteins

Impaired fasting glucose: Baseline FBG 100–125 or A1c 5.6–6.4

^a^missing for 39% donors and 0.6% of non-donors

^b^missing for 34% donors and 0.6% of non-donors

^c^missing for 11% of donors and 0% of non-donors

### Evaluation of diabetes development

#### Diabetes incidence

Within the cohort matched on baseline characteristics only, 8% (57/688) of donors and 27% (185/688) of non-donors developed diabetes over median follow-up of 9.6 (interquartile range (IQR): 5.3–14.0) years and 13.6 (IQR: 6.1–20.4) years, respectively. Comparison of donors and non-donors who developed diabetes are included in S13 Table. Within the cohort matched on baseline characteristics plus diabetes-specific risk factors, 9% (15/165) of donors and 24% (39/165) non-donors developed diabetes over median follow-up of 8.8 (IQR: 6.0–12.9) years and 14.9 (IQR: 6.1–21.2) years, respectively. Given differential follow-up between donors and non-donors, follow-up was truncated at 10 years to avoid biasing outcome realization.

#### 10-Year diabetes-free survival

**Fig 1:**
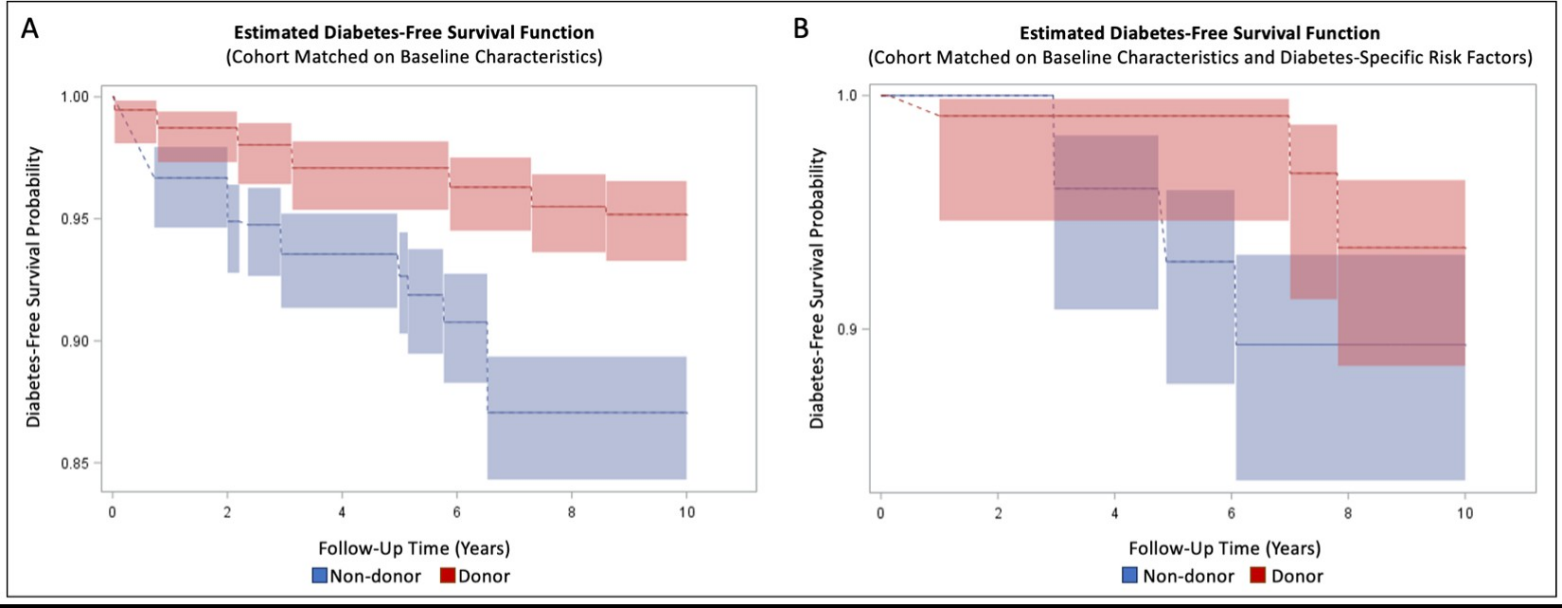
Non-parametric estimated survival models for 10-year diabetes-free survival. A) Diabetes-free survival curves among cohort matched on baseline characteristics only (10-year failure %: Donors 4.8% (95% CI: 3.4%-6.7%); Non-donors 12.9% (95% CI: 10.6%-15.7%); long rank p < .0001). B) Diabetes-free survival curves among cohort matched on baseline characteristics and diabetes-specific risk factors (10-year failure %: Donors 6.5% (95% CI: 3.6%-11.6%); Non-donors 10.7% (95% CI: 6.8%-16.5%); log rank p = 0.03). Shaded area designates 95% confidence limits.

Within the cohort matched on baseline characteristics only, 27/688 (4%) donors and 80/688 (12%) non-donors developed diabetes within 10 years (median follow-up 9.0 (IQR: 5.0–10.0) and 9.3 (IQR: 6.0–10.0) years, respectively). Comparison of donor and non-donor survival curves via log-rank test demonstrated significantly longer time to diabetes development among donors within the first 10 years post-donation (p<0.001; Fig 1A).

Within the cohort matched on baseline characteristics plus diabetes-specific risk factors, 7/165 (4%) donors and 15/165 (9%) non-donors developed diabetes within 10 years (median follow-up 8.5 (IQR: 5.6–10.0) and 9.1 (IQR: 5.9–10.0) years, respectively). Diabetes-free survival was significantly longer among donors within the first decade post-donation (log rank p = 0.03; Fig 1B).

#### Accelerated failure time modeling to assess 10-Year diabetes-free survival

In the cohort matched on baseline characteristics only, donors were estimated to survive 4.2 times longer diabetes-free than non-donors (estimate 4.16; 95% Confidence Interval (CI): 2.08–8.32, p < .001; Table 4). After adjusting for family history of diabetes, impaired fasting glucose, and smoking history, donors trended toward living 2 times longer diabetes-free than non-donors (estimate 1.95; 95% CI: 0.83–4.62, p = 0.13). In this analysis, neither family history of diabetes (estimate 1.02; 95% CI: 0.46–2.26, p = 0.97) nor smoking history (estimate 0.53; 95% CI: 0.24–1.17, p = 0.11) were associated with 10-year diabetes-free survival, but individuals with impaired fasting glucose were estimated to develop diabetes 76% earlier than individuals without impaired fasting glucose (estimate 0.24; 95% CI: 0.09–0.59, p = 0.002). After matching on baseline characteristics plus diabetes-specific risk factors, donors were estimated to live 2 times longer diabetes-free in the first decade post-donation, though this association was still not significant (estimate 1.91; 95% CI: 0.75–4.64, p = 0.15).

**Table 4:** Weibull accelerated failure time model for association of donor status with diabetes onset in matched cohorts excluding donors with only SRTR data, follow-up censored at 10 years.

	Matched on Baseline Characteristics^a^			Matched on Baseline Characteristics^a^			Matched on Baseline Characteristics and Diabetes-Specific Risk Factors^b^		
	**Estimate**	**95%CI**	**p-value**	**Estimate**	**95%CI**	**p-value**	**Estimate**	**95%CI**	**p-value**
**Donor (vs. Non-Donor)**	4.16	2.08–8.32	<0.001	1.95	0.83–4.62	0.13	1.91	0.79–4.64	0.15
**Family history of diabetes**				1.02	0.46–2.26	0.97			
**Impaired fasting glucose**				0.24	0.09–0.59	0.002			
**Ever smoker**				0.53	0.24–1.17	0.11			
**Shape**	0.72	0.57–0.90		0.85	0.82–1.70		0.92	0.56–1.50	
**Observations**	1376			440			330		

^a^Baseline characteristics included age, sex, race, body mass index, systolic and diastolic blood pressure at baseline

^b^Diabetes-specific risk factors included family history of diabetes, impaired fasting glucose, and ever smoker at baseline

Abbreviations: CI = confidence interval

## Discussion

This national study is the first to our knowledge, to compare diabetes development among LKDs and non-donors with obesity, affording the only approximation of donation-attributable risk of diabetes. After matching LKDs to non-donors on baseline characteristics (age, sex, race, BMI, SBP, DBP) plus diabetes-specific risk factors (family history of diabetes, impaired fasting glucose, and smoking history), LKDs with obesity were estimated to live diabetes-free 2 times longer than non-donor counterparts within the first post-donation decade. While this finding was likely not significant due to the limited sample size, these findings suggest there was no increased donation-attributable risk of diabetes among obese donors in the first 10 years post-donation.

Few studies have previously evaluated diabetes development among LKDs. Rates of post-donation diabetes among cohorts of LKDs with median BMIs <28 kg/m^2^ were <5% within 10–15 years post-donation [20–22]. One study stratified LKDs by fasting plasma glucose at donation, and demonstrated that compared to LKDs with normal fasting plasma glucose, LKDs with impaired fasting plasma glucose had 65% higher likelihood of diabetes development [22]. Comparisons of LKDs with and without obesity demonstrated that LKDs with obesity had 2–3 fold higher likelihood of diabetes development compared to non-obese counterparts, and among LKDs with obesity, diabetes incidence ranged from 8–11% over median follow-up periods greater than 10 years [18, 23]. Not surprisingly, the incidence of diabetes among LKDs with obesity in our cohort was higher than that of studies among largely non-obese LKDs, but comparable to that of obese LKDs from Ibrahim et al. when full follow-up was utilized (9% within median 9 years post-donation) [18]. We also found impaired fasting glucose was significantly associated with earlier diabetes development independent of donor status. Our work adds to the existing literature given that more transplant centers and a larger proportion of non-white donors are represented among our LKD cohort compared to these prior studies. To our knowledge, it is also the first study to compare diabetes development among LKDs with obesity and non-donors with obesity, which provides greater understanding of the association between donation itself and diabetes development within a population at greater baseline risk of both diabetes and ESKD [17, 34, 35].

Rather than suggesting that donation may have a protective effect against diabetes, it may be argued that differences between the donors and non-donors utilized in this study exist despite the application of donor eligibility criteria to non-donor records [31]. Prior studies have utilized CARDIA and ARIC participants as non-donor controls [36, 37], though diabetes development between LKDs and non-donors with obesity has not been evaluated. As such, it is possible that intensive donor evaluations select for the 10–20% of persons with obesity that are metabolically healthy and thus at lower risk for diabetes [19, 38]. Metabolically healthy obesity has been defined as a BMI ≥30.0 kg/m^2^ in the absence of any cardiometabolic abnormalities that comprise metabolic syndrome [38, 39]. In this study, we matched on a majority of these cardiometabolic factors including BMI, SBP, DBP and impaired fasting glucose. Triglycerides did not significantly differ between donors and non-donors in matched cohorts, and only small clinical differences in HDL were noted. Despite this, residual confounding attributable to the donor evaluation is possible. Metabolically healthy obesity has been associated with phenotypic characteristics not captured in this study, but which may have been assessed during a donor evaluation, such as lower visceral fat and decreased hepatic steatosis via computed tomography imaging, or greater lower extremity adiposity compared to central adiposity and maintained cardiopulmonary fitness evaluated during physical exam [38]. However, important heath-associated attributes specific to the non-donors should also be considered as a result of volunteer bias. The non-donors from CARDIA and ARIC comprise a select group of participants who enrolled in a longitudinal study and have demonstrated adherence to decades-long follow-up [29, 30, 34]. In contrast, 2-year post-donation follow-up remains challenging among living kidney donors [40].

A second possible explanation of our findings is that donors may become more health conscious following donation to protect their remaining kidney. In a qualitative evaluation that explored LKDs’ perceived health benefits, a donor reported increased physical activity or regular follow-up with a healthcare provider following donation, suggesting the act of donation may alter an individual’s health trajectory [41]. However, donation-associated health consciousness has not been studied in larger LKD populations and may not be generalizable. Other literature suggests donors gain weight with aging like the general population, suggesting health-conscious behaviors among donors and non-donors may be similar [23, 42]. Recent work by our group has demonstrated that weight gain among donors is associated with greater risk of hypertension than that observed among non-donors experiencing similar weight gain [43]. Consequently, there may be a subset of donors with obesity who are also at greater risk of diabetes than comparable non-donors, although the low event rate in the current study precluded our ability to incorporate longitudinal BMI measurements. Conservatively, donors with obesity should be counseled on the importance of maintaining a healthy weight post-donation, particularly if other known risk factors for diabetes, as shown in this study, are present, as hyperinsulinemia has been associated with glomerular hyperfiltration and increased vascular permeability [44]. Future work in a larger cohort is necessary to explore the role of BMI trajectory in modifying diabetes risk, in addition to direct comparisons of healthcare utilization and health consciousness of donors and non-donors.

A final possible explanation for longer diabetes-free survival among LKDs with obesity in the first decade post-donation may be rooted in altered insulin and glucose metabolism following donor nephrectomy. A recent prospective study from Tanriover et al. evaluated insulin sensitivity pre-donation and 3 months post-donation among nine LKDs who demonstrated significantly greater insulin, but similar glucose concentrations, and lower measures of insulin sensitivity post-donation [25]. Notably, the decreases in insulin sensitivity were greater among four LKDs with obesity [25]. Understanding hyperinsulinemia fosters insulin resistance [45], but insulin resistance may persist for approximately a decade prior to the development of pancreatic beta-cell insufficiency [46], the implications of these data are twofold. First, post-donation hyperinsulinemia may prevent the detection of hyperglycemia required to diagnose diabetes during the first decade post-donation. This is consistent with the findings in the present study. Second, however, LKDs may be at greater risk for diabetes development compared to non-donors in subsequent decades due to a greater degree and/or earlier development of insulin resistance. The small sample studied in Tanriover et al. is a limitation yet provides further evidence of the paucity of data regarding the metabolic implications of living kidney donation [25]. Should the findings be replicated in a larger cohort, it would further emphasize the critical importance of longitudinal studies of LKDs with obesity to adequately assess diabetes development in later decades.

Important limitations of this study should be acknowledged. First, while baseline BMI was utilized for cohort matching, we were unable to account for BMI trajectory over the follow-up period. While unlikely given literature suggests LKDs gain weight over time, differential weight changes among donors relative to non-donors could bias our results [42]. Second, participants were enrolled during differing eras amid increasing secular trends in obesity and diabetes prevalence, though limiting our study population to persons with obesity precluded the need to control for enrollment year [35, 47]. Third, both non-donor cohorts had standardized follow-up at predetermined intervals given prospective data collection while donors were not systematically tested for diabetes given retrospective data collection, which may bias outcome ascertainment. However, while research data may be more systematically captured, median number of healthcare encounters per post-donation follow-up year among donors suggested more frequent follow-up on average than non-donors and thus more encounters at which to assess disease development (S3 Table). Fourth, follow-up time among donors and non-donors was truncated at 10 years. Transplant centers have become more aggressive in acceptance of living donors with isolated medical abnormalities, and it is possible that more marginal donors in recent years have not had enough time since donation to develop new-onset disease. While truncating follow-up at 10 years inherently limited time for outcome realization, it afforded comparable follow-up times to avoid biasing our results. Fifth, there were limitations regarding diabetes-specific risk factors among donors and non-donors. Family history of diabetes among non-donors may be under-ascertained given that data were collected when participants, and their family members, were young. Among donors, there was substantial missing data for diabetes-specific risk factors, which limited the sample-size of our complete case analysis. However, sensitivity analyses after multiply imputing for diabetes-specific risk factors support the inferences of our primary analyses (S9 Table). The missingness in our data emphasize the complex nature of data collection among LKDs for whom post-donation follow-up is only required for 2 years and is known to be poor [40]. Robust data capture of information including but not limited to anthropomorphic measurements (height, weight, waist-to-hip ratio), blood pressure, comprehensive metabolic panels, lipids, and psychosocial outcomes for well beyond the minimal two year requirement is paramount for improving post-donation risk prediction. Finally, this was a retrospective, observational study which only allows for the evaluation of associations. More clinical and translational work is needed to better elucidate explanations of our findings.

This study utilized a national cohort of LKDs with obesity and to our knowledge is the first to compare diabetes development between donors and non-donors, providing the only approximation of donation-attributable diabetes risk. After matching LKDs to non-donors on baseline characteristics and diabetes-specific risk factors, donors trended toward living longer diabetes-free compared to non-donors within the first decade post-donation. While our data suggest donation-attributable risk of diabetes among living donors with obesity may be negligible within 10 years post-donation, more longitudinal work is required to evaluate diabetes risk long-term, to prioritize safety in the development of evidence-based selection criteria for LKDs with obesity.

## Supporting information

S1 File (PDF)

S2 File (PDF)

S1 Table Non-mutually exclusive sources of family history of diabetes among donors and non-donors within the cohort matched on baseline characteristics. (PDF)

S2 Table Alcohol use among donors and non-donors matched on baseline characteristics. (PDF)

S3 Table Total number of post-donation healthcare encounters and time since donation among all donors included in study. (PDF)

S4 Table Diabetes diagnosis by source among donors and non-donors matched on baseline characteristics. (PDF)

S5 Table History of gestational diabetes at baseline among female donors and CARDIA non-donors from cohort matched on baseline characteristics. (PDF)

S6 Table Distribution of age and study exam year among CARDIA and ARIC non-donor records matched to donors on baseline characteristics. (PDF)

S7 Table Interval censored Cox Proportional Hazards models for 10-year risk of diabetes development among unique donors and non-donors. (PDF)

S8 Table Weibull accelerated failure time model for association of donor status with diabetes onset in matched cohorts including donors with only SRTR data, follow-up censored at 10 years. (PDF)

S9 Table Weibull accelerated failure time model for association of donor status with diabetes onset in matched cohorts following multiple imputation of diabetes-specific risk factors, follow up censored at 10 years. (PDF)

S10 Table Weibull accelerated failure time model for association of donor status with diabetes onset, follow up censored at 10 years, utilizing last non-donor record by age to identify unique individuals. (PDF)

S11 Table Weibull accelerated failure time model for association of donor status with diabetes onset in matched cohorts among donors and non-donors with eGFR ≥80 mL/min, excluding donors with only SRTR data, follow-up censored at 10 years. (PDF)

S12 Table Weibull accelerated failure time model for association of donor status with diabetes onset in matched cohorts excluding donors with only SRTR data and utilizing full follow-up. (PDF)

S13 Table Demographics and baseline characteristics of unique living kidney donors (1R01DK113980 and 1R01096008) and non-donors (CARDIA and ARIC) who developed diabetes from the cohort matched on baseline characteristics. (PDF)

S1 Fig CONSORT diagram for living kidney donors. (PDF)

S2 Fig CONSORT diagram for CARDIA non-donors. (PDF)

S3 Fig CONSORT diagram for ARIC non-donors. (PDF)

S4 Fig Non-parametric estimated survival models for diabetes-free survival utilizing full follow-up among cohort matched on baseline characteristics only. (PDF)
