# Supplementary material for: Diabetes-free survival among living kidney donors and non-donors with obesity: A longitudinal cohort study
Source: PLoS One. 2022 Nov 18;17(11):e0276882. doi: 10.1371/journal.pone.0276882 (PMC9674148; doi:10.1371/journal.pone.0276882)
Supplement: S1 File — (PDF) [file pone.0276882.s001.pdf]

Supplemental Methods

Data Collection Among Living Kidney Donors with Obesity

Upon initial contact with the donor, basic data such as name, date of birth, date of transplant and transplant center are verified, and additional demographic data is obtained including sex, gender, race, and ethnicity.

Consenting participants are asked to complete an initial survey (and annually thereafter for four years) that collects data on additional demographics, socioeconomics, self-reported medical history and psychosocial history.

Among participants who completed medical release forms, records from the transplant center at which they donated and their primary care provider are obtained. Medical records from the transplant center include the donor evaluation with accompanying labs and CT imaging, the operative report from the living donor nephrectomy, records from the post-operative hospital stay and any post-donation follow-up labs. Medical records from the primary care provider are requested from post-operative day 1 to the present date. Each subsequent year, records are requested from the date of the last set of records to the present date. Data obtained from the primary care provider medical records include diagnosis of diabetes, HgbA1c, fasting glucose, urine studies, and medications.

Additional Information Regarding Exposures

Family history of diabetes among donors was defined as any reported family history to include parents, siblings, aunts, uncles, grandparents, cousins, and nieces/nephews. Family history among non-donors was defined as any reported family history to include parents and siblings (Table S1).

For non-donors, CARDIA prospectively captured alcohol use as “had a drink in the past year” and ARIC prospectively captured alcohol use as “ever drinker.” For donors, alcohol use was retrospectively collected from the time of evaluation

and obtained from the EMR. Given that these data are in inherently different and not comparable, we were unable to account for alcohol use in our primary analyses though these data are provided in Table S2.

Sensitivity Analyses

In each of the additional analyses described below, three models were created and utilized diabetes development within 10 years from baseline as the outcome. The first utilized a cohort matched on baseline characteristics and evaluated donor status as the exposure. The second model utilized this same cohort matched on baseline characteristics and evaluated donor status as the exposure while controlling for the diabetes-specific risk factors (family history of diabetes, impaired fasting glucose, and history of ever smoking). The third model utilized a cohort matched on baseline characteristics plus the diabetes-specific risk factors and evaluated donor status as the exposure.

Given that accelerated failure time (AFT) models are used less frequently in our field, the three models described above were also fit using Cox proportional hazards methods on interval censored data (Table S7).

For a more robust evaluation of early diabetes development, we included donors with only SRTR data for whom follow-up was limited to median 1.98 years (N=983). Matching as described in the main paper methods and the AFT models as described above were repeated (Table S8).

Due to substantial missing data in the diabetes-specific risk factors described above, multiple imputation was performed under the assumption that data were missing at random. In total, 20 datasets were imputed using the method of predictive mean matching to impute missing values. Variables used in the multiple imputation model included donor status, age, gender, year, baseline SBP, baseline DBP, new diabetes onset, baseline BMI, serum creatinine, eGFR, data source, white race, family history of hypertension, family history of diabetes, fasting blood glucose, impaired glucose, baseline HDL, baseline triglycerides, cholesterol, and history of ever smoking. After imputing the 20 datasets, matching

and cohort creation was implemented within each imputation. AFT models were subsequently performed as described above with estimates and standard errors combined using Rubin's Rules (Table S9).(1)

Given that the cohorts utilized in our primary analyses were limited to unique individuals' earliest matched record by age, cohorts were also created utilizing each unique individuals' latest matched record by age. The three models described above were repeated (Table S10).

Given that prior guidelines recommended an eGFR threshold of 80 mL/min,(2) we performed a sensitivity analysis in which donors and non-donors were excluded for eGFR < 80 mL/min. Matching on baseline characteristics, and baseline characteristics plus diabetes-specific risk factors, was repeated and the three models described above were performed (Table S11).

Given that CARDIA and ARIC had standardized follow-up with exceptional adherence among their participants, our non-donors had significantly longer follow-up time compared to donors (median years follow-up among non-donors matched to donors on baseline characteristics plus diabetes-specific risk factors (IQR): 14.7 (6.1, 21.1 vs. 8.7 (6.1, 12.9) p<.001). Thus, to avoid biasing ascertainment of diabetes between donors and non-donors, follow-up time was truncated at 10 years to achieve comparable follow-up between groups for matched analyses. However, as a sensitivity analysis, we evaluated diabetes-free survival between donors and non-donors utilizing full follow-up among the cohort matched on baseline characteristics. The survival curve is included as Figure S4. Additionally, the three models described above were also repeated (Table S12).

The R package MatchIt (ver. 4.1.0) was used to match donor and non-donor records. We used SAS proc iclifetest for non-parametric analysis of survival data, proc icphreg for Cox proportional hazards models with interval censored data, and proc lifereg for estimation of AFT models. Multiple imputation was performed with the package mice (ver. 3.13.0).
