# Supplementary material for: Diabetes-free survival among living kidney donors and non-donors with obesity: A longitudinal cohort study
Source: PLoS One. 2022 Nov 18;17(11):e0276882. doi: 10.1371/journal.pone.0276882 (PMC9674148; doi:10.1371/journal.pone.0276882)
Supplement: S2 File — (PDF) [file pone.0276882.s002.pdf]

Supplemental References

1. Rubin D: Multiple Imputation for Nonresponse in Surveys. In: *Wiley Online Library.* 1987,
2. Kidney Disease: Improving Global Outcomes (KDIGO) Living Kidney Donor Work Group. KDIGO Clinical Practice Guideline on the Evaluation and Care of Living Kidney Donors. *Transplantation,* 101**:** S1–S109, 2017
