# Supplementary material for: Diabetes-free survival among living kidney donors and non-donors with obesity: A longitudinal cohort study
Source: PLoS One. 2022 Nov 18;17(11):e0276882. doi: 10.1371/journal.pone.0276882 (PMC9674148; doi:10.1371/journal.pone.0276882)
Supplement: S1 Table — (PDF) [file pone.0276882.s003.pdf]

Diabetes-Free Survival Among Living Kidney Donors and Non-Donors with Obesity: A Longitudinal Cohort Study

Table S1. Non-mutually exclusive sources of family history of diabetes among donors and non-donors within the cohort matched on baseline characteristics (N=1376)

	No family history of diabetes	Maternal history of diabetes	Paternal history of diabetes	Other family history of diabetes	Missing family history
Donor	286 (41.6)	70 (10.2)	69 (10.0)	119 (17.3)	189 (27.4)
Non-donor	367 (53.3)	100 (14.5)	73 (10.6)	66 (9.6)	139 (20.2)
