# Supplementary material for: Diabetes-free survival among living kidney donors and non-donors with obesity: A longitudinal cohort study
Source: PLoS One. 2022 Nov 18;17(11):e0276882. doi: 10.1371/journal.pone.0276882 (PMC9674148; doi:10.1371/journal.pone.0276882)
Supplement: S2 Table — (PDF) [file pone.0276882.s004.pdf]

Diabetes-Free Survival Among Living Kidney Donors and Non-Donors with Obesity: A Longitudinal Cohort Study

Table S2. Alcohol use among donors and non-donors matched on baseline characteristics (N=1376)

	Donors (N=688)	CARDIA Non-donors (N=288)	ARIC Non-donors (N=400)
Ever drinker	234 (34.0)		296 (74.0)
Never drinker	238 (34.6)		104 (26.0)
Drink in past year		227 (78.8)	
No drink in past year		57 (19.8)	
Missing information on alcohol use	216 (31.4)	4 (1.4)	
