# Supplementary material for: Diabetes-free survival among living kidney donors and non-donors with obesity: A longitudinal cohort study
Source: PLoS One. 2022 Nov 18;17(11):e0276882. doi: 10.1371/journal.pone.0276882 (PMC9674148; doi:10.1371/journal.pone.0276882)
Supplement: S3 Table — (PDF) [file pone.0276882.s005.pdf]

Table S3. Total number of post-donation healthcare encounters and time since donation among all donors included in study (N=1119)

	Median (IQR)
Number of post-donation blood pressure measurements	6 (3-12)
Time from donation to measurement (years)	6.3 (2.1-11.4)
Number of post-donation glucose measurements	4 (2-7)
Time from donation to measurement (years)	5.6 (0.8-10.9)
