# Supplementary material for: Diabetes-free survival among living kidney donors and non-donors with obesity: A longitudinal cohort study
Source: PLoS One. 2022 Nov 18;17(11):e0276882. doi: 10.1371/journal.pone.0276882 (PMC9674148; doi:10.1371/journal.pone.0276882)
Supplement: S4 Table — (PDF) [file pone.0276882.s006.pdf]

Table S4. Diabetes diagnosis by source among donors and non-donors matched on baseline characteristics.

Donor Status	SRTR (N=0)	EMR (N=31)	Reported use of diabetes medication (N=23)	A1c (N=1)	Consecutive random glucose measurements (N=5)	Self-report (N=215)	Single elevated fasting glucose (N=127)	N (N=1376)
Donor	-	1	1	1	-	1	-	1
Donor	-	1	1	-	-	1	-	8
Donor	-	1	1	-	-	-	-	1
Donor	-	1	-	-	1	-	-	1
Donor	-	1	-	-	-	1	-	9
Donor	-	1	-	-	-	-	-	11
Donor	-	-	1	-	1	1	-	1
Donor	-	-	1	-	-	1	-	8
Donor	-	-	1	-	-	-	-	4
Donor	-	-	-	-	1	1	-	1
Donor	-	-	-	-	-	1	-	12
Donor	-	-	-	-	-	-	-	631
Non-donor	-	-	-	-	1	1	1	2
Non-donor	-	-	-	-	-	1	1	115
Non-donor	-	-	-	-	-	1	-	58
Non-donor	-	-	-	-	-	-	1	10
Non-donor	-	-	-	-	-	-	-	503
