# Supplementary material for: Diabetes-free survival among living kidney donors and non-donors with obesity: A longitudinal cohort study
Source: PLoS One. 2022 Nov 18;17(11):e0276882. doi: 10.1371/journal.pone.0276882 (PMC9674148; doi:10.1371/journal.pone.0276882)
Supplement: S5 Table — (PDF) [file pone.0276882.s007.pdf]

Table S5. History of gestational diabetes at baseline among female donors and CARDIA non-donors from cohort matched on baseline characteristics.

	Female Donor (N=433)	CARDIA Female Non-donor* (N=177)
History of gestational diabetes	3 (0.7)	2 (1.1)
No evidence of gestational diabetes	430 (99.3)	175 (98.9)

*Not captured in ARIC
