# Supplementary material for: Diabetes-free survival among living kidney donors and non-donors with obesity: A longitudinal cohort study
Source: PLoS One. 2022 Nov 18;17(11):e0276882. doi: 10.1371/journal.pone.0276882 (PMC9674148; doi:10.1371/journal.pone.0276882)
Supplement: S6 Table — (PDF) [file pone.0276882.s008.pdf]

Table S6. Distribution of age and study exam year among CARDIA and ARIC non-donor records matched to donors on baseline characteristics.

	ARIC Baseline Exam (N=252)	ARIC 3-year Exam (N=113)	ARIC 9-year Exam (N=35)	CARDIA Baseline Exam (N=57)	CARDIA 10-year Exam (N=74)	CARDIA 15-year Exam (N=66)	CARDIA 20-year Exam (N=59)	CARDIA 25-year Exam (N=32)	Total (N=688)
Age (mean (SD))	51.865 (5.322)	55.540 (5.125)	59.629 (4.839)	26.649 (2.800)	35.446 (3.597)	40.167 (3.837)	44.153 (3.503)	47.812 (3.930)	47.036 (10.183)
