# Supplementary material for: Diabetes-free survival among living kidney donors and non-donors with obesity: A longitudinal cohort study
Source: PLoS One. 2022 Nov 18;17(11):e0276882. doi: 10.1371/journal.pone.0276882 (PMC9674148; doi:10.1371/journal.pone.0276882)
Supplement: S7 Table — (PDF) [file pone.0276882.s009.pdf]

Table S7. Interval censored Cox Proportional Hazards models for 10-year risk of diabetes development among unique donors and non-donors

	Matched on Baseline Characteristics[a]			Matched on Baseline Characteristics[a]			Matched on Baseline Characteristics and Diabetes-Specific Risk Factors[b]		
	**HR**	**95% CI**	**p-value**	**HR**	**95% CI**	**p-value**	**HR**	**95% CI**	**p-value**
**Donor (vs. Non-Donor)**	0.36	0.23 - 0.55	<0.001	0.56	0.28 – 1.14	00.11	0.49	0.20 – 1.21	0.04
**Family history of diabetes**				0.98	0.50 – 1.94	0.96			
**Impaired fasting glucose**				3.43	1.77 – 6.62	<.001			
**Ever smoker**				1.73	0.91 – 3.29	0.09			
**Observations**	1376			440			330		

[a]Baseline characteristics included age, sex, race, body mass index, systolic and diastolic blood pressure at baseline
[b]Diabetes-specific risk factors included family history of diabetes impaired fasting glucose, and smoking history at baseline
Abbreviations: HR = hazard ratio; CI = confidence interval
