# Supplementary material for: Diabetes-free survival among living kidney donors and non-donors with obesity: A longitudinal cohort study
Source: PLoS One. 2022 Nov 18;17(11):e0276882. doi: 10.1371/journal.pone.0276882 (PMC9674148; doi:10.1371/journal.pone.0276882)
Supplement: S8 Table — (PDF) [file pone.0276882.s010.pdf]

Table S8. Weibull accelerated failure time model for association of donor status with diabetes onset in matched cohorts including donors with only SRTR data, follow-up censored at 10 years.

	**Matched on Base Characteristics[a]**			**Matched on Base Characteristics[a]**			**Matched on Base Characteristics and Diabetes-Specific Risk Factors[b]**		
	**Estimate**	**95% CI**	**p-value**	**Estimate**	**95% CI**	**p-value**	**Estimate**	**95% CI**	**p-value**
**Donor (vs. Non-Donor)**	4.61	2.19 – 14.48	<0.001	2.80	1.03 – 7.61	0.05	2.44	0.81 – 7.41	0.11
**Family History of diabetes**				1.16	0.49 – 2.71	0.74			
**Impaired fasting glucose**				0.10	0.03 – 0.33	<0.001			
**Ever smoker**				0.65	0.29 – 1.44	0.29			
**Shape**	0.78	0.65 – 0.94		0.76	0.54 – 1.07		0.88	0.54 – 1.43	
**Observations**									

[a]Baseline characteristics included age, sex, race, body mass index, systolic and diastolic blood pressure at baseline
[b]Diabetes-specific risk factors included family history of diabetes impaired fasting glucose, and smoking history at baseline
Abbreviations: CI = confidence interval
