# Supplementary material for: Diabetes-free survival among living kidney donors and non-donors with obesity: A longitudinal cohort study
Source: PLoS One. 2022 Nov 18;17(11):e0276882. doi: 10.1371/journal.pone.0276882 (PMC9674148; doi:10.1371/journal.pone.0276882)
Supplement: S9 Table — (PDF) [file pone.0276882.s011.pdf]

Table S9. Weibull accelerated failure time model for association of donor status with diabetes onset in matched cohorts following multiple imputation of diabetes-specific risk factors, follow up censored at 10 years.

	*Matched on Baseline Characteristics[a]			Matched on Baseline Characteristics[a]			Matched on Baseline Characteristics and Diabetes-Specific Risk Factors[b]		
	**Estimate**	**95% CI**	**p-value**	**Estimate**	**95% CI**	**p-value**	**Estimate**	**95% CI**	**p-value**
**Donor (vs. Non-Donor)**				3.37	1.18 – 5.56	<0.001	3.16	2.08 – 4.71	0.01
**Family history of diabetes**				0.56	0.33 – 0.94	0.03			
**Impaired fasting glucose**				0.15	0.7 – 0.29	<0.001			
**Ever smoker**				0.53	0.31 – 0.89	0.02			
**Shape**				0.76	0.59 – 0.93		0.75	0.62 – 0.86	
**Mean Observations (M=20 datasets)**				1376			822.2 (SD=18.3)		

[a]Baseline characteristics included age, sex, race, body mass index, systolic and diastolic blood pressure at baseline
[b]Diabetes-specific risk factors included family history of diabetes impaired fasting glucose, and smoking history at baseline
Abbreviations: CI = confidence interval
*Same model as primary analyses given nothing in this model was imputed.
