# Supplementary material for: Diabetes-free survival among living kidney donors and non-donors with obesity: A longitudinal cohort study
Source: PLoS One. 2022 Nov 18;17(11):e0276882. doi: 10.1371/journal.pone.0276882 (PMC9674148; doi:10.1371/journal.pone.0276882)
Supplement: S10 Table — (PDF) [file pone.0276882.s012.pdf]

Table S10. Weibull accelerated failure time model for association of donor status with diabetes onset, follow up censored at 10 years, utilizing last non-donor record by age to identify unique individuals.

	**Matched on Base Characteristics[a]**			**Matched on Base Characteristics[a]**			**Matched on Base Characteristics and Diabetes-Specific Risk Factors[b]**		
	**Estimate**	**95% CI**	**p-value**	**Estimate**	**95% CI**	**p-value**	**Estimate**	**95% CI**	**p-value**
**Donor (vs. Non-Donor)**	4.61	2.30 – 9.23	<.001	3.79	1.1.51 – 9.50	0.002	1.74	0.79 – 3.84	0.17
**Family History of diabetes**				0.55	0.30 – 1.00	0.049			
**Impaired fasting glucose**				0.24	0.12 – 0.48	<.001			
**Ever smoker**				0.47	0.25 – 0.87	0.02			
**Shape**	0.74	0.59 – 0.92		0.77	0.59 – 1.00		2.1.14	0.71 – 1.85	
**Observations**	1376			816			330		

[a]Baseline characteristics included age, sex, race, body mass index, systolic and diastolic blood pressure at baseline
[b]Diabetes-specific risk factors included family history of diabetes impaired fasting glucose, and smoking history at baseline
Abbreviations: CI = confidence interval
