# Supplementary material for: Diabetes-free survival among living kidney donors and non-donors with obesity: A longitudinal cohort study
Source: PLoS One. 2022 Nov 18;17(11):e0276882. doi: 10.1371/journal.pone.0276882 (PMC9674148; doi:10.1371/journal.pone.0276882)
Supplement: S11 Table — (PDF) [file pone.0276882.s013.pdf]

Table S11. Weibull accelerated failure time model for association of donor status with diabetes onset in matched cohorts among donors and non-donors with eGFR ≥80 mL/min, excluding donors with only SRTR data, follow-up censored at 10 years.

	**Matched on Baseline Characteristics[a]**			**Matched on Baseline Characteristics[a]**			**Matched on Baseline Characteristics and Diabetes-Specific Risk Factors[b]**		
	**Estimate**	**95% CI**	**p-value**	**Estimate**	**95% CI**	**p-value**	**Estimate**	**95% CI**	**p-value**
**Donor (vs. Non-Donor)**	5.63	2.19 – 14.48	<0.001	4.76	1.02 – 22.12	0.05	2.00	0.63 – 6.35	0.24
**Family history of diabetes**				0.58	0.16 – 2.02	0.39			
**Impaired fasting glucose**				0.08	0.02 – 0.39	0.002			
**Ever smoked**				0.71	0.22 – 2.32	0.57			
**Shape**	0.65	0.50 – 0.85		0.61	0.40 – 0.91		0.84	0.50 – 1.43	
**Observations**	1100			338			264		

[a]Baseline characteristics included age, sex, race, body mass index, systolic and diastolic blood pressure at baseline
[b]Diabetes-specific risk factors included family history of diabetes, impaired fasting glucose, and smoking at baseline
Abbreviations: CI = confidence interval
