# Supplementary material for: Diabetes-free survival among living kidney donors and non-donors with obesity: A longitudinal cohort study
Source: PLoS One. 2022 Nov 18;17(11):e0276882. doi: 10.1371/journal.pone.0276882 (PMC9674148; doi:10.1371/journal.pone.0276882)
Supplement: S12 Table — (PDF) [file pone.0276882.s014.pdf]

Table S12. Weibull accelerated failure time model for association of donor status with diabetes onset in matched cohorts excluding donors with only SRTR data and utilizing full follow-up.

	**Matched on Baseline Characteristics[a]**			**Matched on Baseline Characteristics[a]**			**Matched on Baseline Characteristics and Diabetes-Specific Risk Factors[b]**		
	**Estimate**	**95% CI**	**p-value**	**Estimate**	**95% CI**	**p-value**	**Estimate**	**95% CI**	**p-value**
**Donor (vs. Non-Donor)**	2.18	1.63 – 2.93	<0.001	1.44	0.98 – 2.12	0.06	1.47	0.91– 2.40	0.12
**Family history of diabetes**				1.00	0.71 – 1.42	0.98			
**Impaired fasting glucose**				0.40	0.28 – 0.57	<0.0001			
**Ever smoker**				0.95	0.68 – 1.32	0.75			
**Shape**	1.13	1.00 – 1.29		1.31	1.07 – 1.60		1.33	1.02 – 1.73	
**Observations**	1376			440			330		

[a]Baseline characteristics included age, sex, race, body mass index, systolic and diastolic blood pressure at baseline
[b]Diabetes-specific risk factors included family history of diabetes, impaired fasting glucose, and ever smoker at baseline
Abbreviations: CI = confidence interval
