# Supplementary material for: Diabetes-free survival among living kidney donors and non-donors with obesity: A longitudinal cohort study
Source: PLoS One. 2022 Nov 18;17(11):e0276882. doi: 10.1371/journal.pone.0276882 (PMC9674148; doi:10.1371/journal.pone.0276882)
Supplement: S13 Table — (PDF) [file pone.0276882.s015.pdf]

Table S13. Demographics and baseline characteristics of unique living kidney donors (1R01DK113980 and 1R01096008) and non-donors (CARDIA and ARIC) who developed diabetes from the cohort matched on baseline characteristics.

	Total with diabetes onset (N=242) N (%)	Donors with diabetes onset (N=57) N (%)	Non-donors with diabetes onset (N=185) N (%)	p-value
Age (years), mean (SD)	47.4 (10.0)	46.0 (9.3)	47.9 (10.2)	0.21
Female Sex	148 (61.2%)	34 (59.6%)	114 (61.6%)	0.79
Race				0.19
Non-White	49 (20.2%)	15 (26.3%)	34 (18.4%)	
White	193 (79.8%)	42 (73.7%)	151 (81.6%)	
BMI (kg/m^2), mean (SD)	32.9 (2.3)	33.4 (2.414)	32.8 (2.3)	0.08
WHO class				0.38
Class I (30-34.9)	197 (81.4%)	44 (77.2%)	153 (82.7%)	
Class II (35-39.9)	41 (16.9%)	11 (19.3%)	30 (16.2%)	
Class III (40+)	4 (1.7%)	2 (3.5%)	2 (1.1%)	
SBP (mmHg), mean (SD)	121.2 (9.7)	121.5 (10.9)	121.2 (9.3)	0.80
DBP (mmHg), mean (SD)	74.1 (7.3)	74.6 (6.8)	73.9 (7.4)	0.52
Serum Creatinine, mean (SD)	0.75 (0.16)	0.85 (0.18)	0.71 (0.14)	< 0.001
eGFR, mean (SD)	105.1 (14.2)	96.8 (16.4)	107.7 (12.4)	< 0.001
HDL[a]	45.2 (13.7)	48.7 (10.4)	45.0 (13.9)	0.44
Triglycerides[b]	151.8 (90.9)	174.2 (93.6)	150.4 (90.8)	0.38
History of high cholesterol				0.50
No	94 (88.7%)	41 (91.1%)	53 (86.9%)	
Yes	12 (11.3%)	4 (8.9%)	8 (13.1%)	
Missing	136	12	124	
Ever smoker				0.02
No	129 (55.1%)	34 (69.4%)	95 (51.4%)	
Yes	105 (44.9%)	15 (30.6%)	90 (48.6%)	
Missing	8	8	0	
Fasting blood glucose[c]	102.6 (12.9)	103.1 (19.6)	102.5 (11.4)	0.81
Impaired fasting glucose				0.55
No	87 (41.6%)	14 (46.7%)	73 (40.8%)	
Yes	122 (58.4%)	16 (53.3%)	106 (59.2%)	
Missing	33	27	6	
Family history of diabetes				0.53
No	128 (61.2%)	30 (65.2%)	98 (60.1%)	
Yes	81 (38.8%)	16 (34.8%)	65 (39.9%)	
Missing	33	11	22	
Family history of hypertension				0.003
No	103 (46.8%)	30 (66.7%)	73 (41.7%)	
Yes	117 (53.2%)	15 (33.3%)	102 (58.3%)	

Missing	22	12	10	

Abbreviations: SD = standard deviation; BMI = Body Mass Index; WHO = World Health Organization; SBP = Systolic blood pressure; DBP = Diastolic blood pressure; eGFR = estimated glomerular filtration rate; HDL = high density lipoproteins

Impaired fasting glucose: Baseline FBG 100-125 or A1c 5.6-6.4

[a]missing for 84% of donors and <1% of non-donors

[b]missing for 79% of donors and <1% of non-donors

[c]missing for 46% of donors and 3% of non-donors
