# Supplementary material for: Diabetes-free survival among living kidney donors and non-donors with obesity: A longitudinal cohort study
Source: PLoS One. 2022 Nov 18;17(11):e0276882. doi: 10.1371/journal.pone.0276882 (PMC9674148; doi:10.1371/journal.pone.0276882)

Diabetes-Free Survival Among Living Kidney Donors and Non-Donors with Obesity: A Longitudinal Cohort Study

Figure S1. CONSORT diagram for living kidney donors

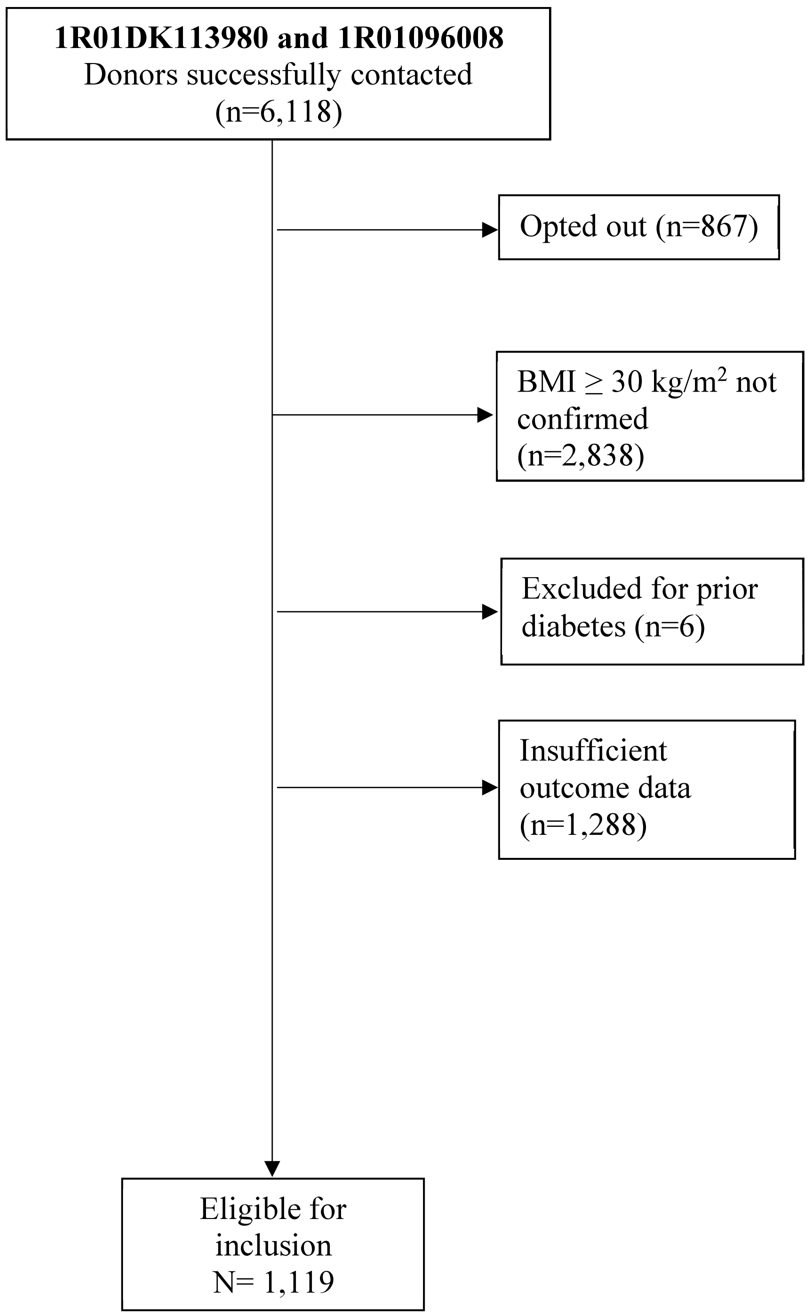

Supplement: S1 Fig — (PDF) [file pone.0276882.s016.pdf]
