# Supplementary figures and images for: Diabetes-free survival among living kidney donors and non-donors with obesity: A longitudinal cohort study
Source: PLoS One. 2022 Nov 18;17(11):e0276882. doi: 10.1371/journal.pone.0276882 (PMC9674148; doi:10.1371/journal.pone.0276882)

Figure S2. CONSORT diagram for CARDIA

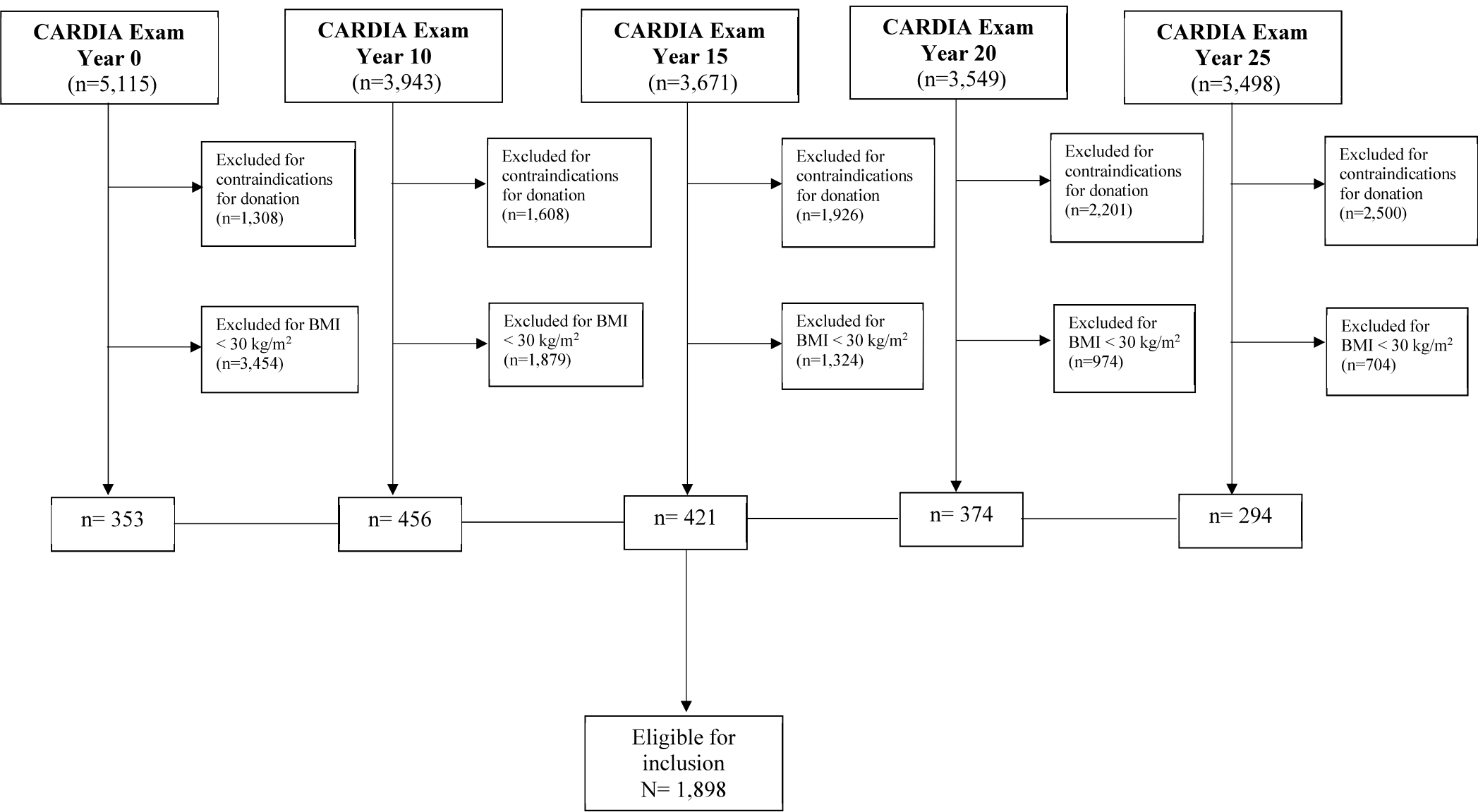

Supplement: S2 Fig — (PDF) [file pone.0276882.s017.pdf]

Figure S3. CONSORT diagram for ARIC

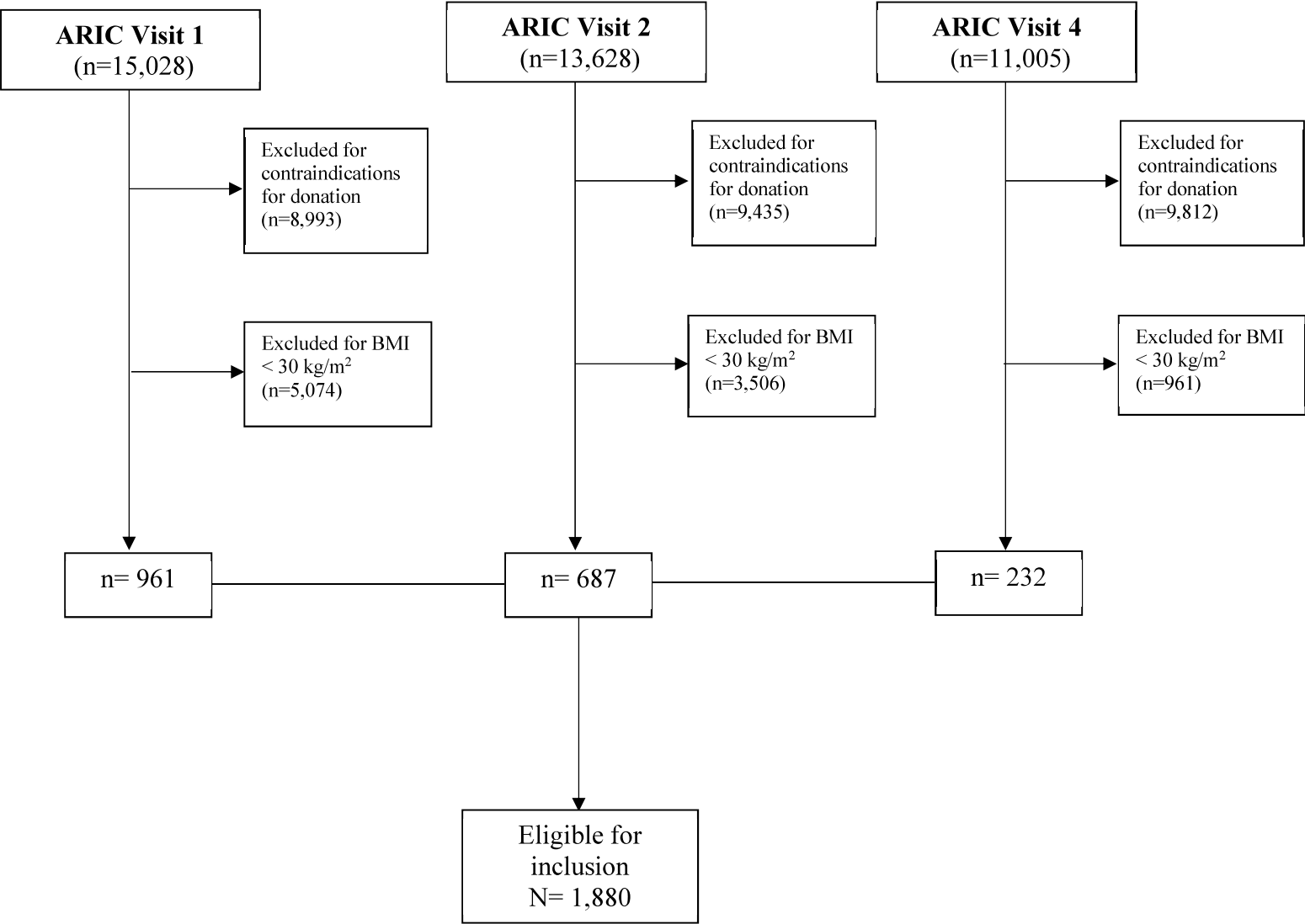

Supplement: S3 Fig — (PDF) [file pone.0276882.s018.pdf]
