# Supplementary material for: Diabetes-free survival among living kidney donors and non-donors with obesity: A longitudinal cohort study
Source: PLoS One. 2022 Nov 18;17(11):e0276882. doi: 10.1371/journal.pone.0276882 (PMC9674148; doi:10.1371/journal.pone.0276882)
Supplement: S4 Fig — (PDF) [file pone.0276882.s019.pdf]

Diabetes-Free Survival Among Living Kidney Donors and Non-Donors with Obesity: A Longitudinal Cohort Study

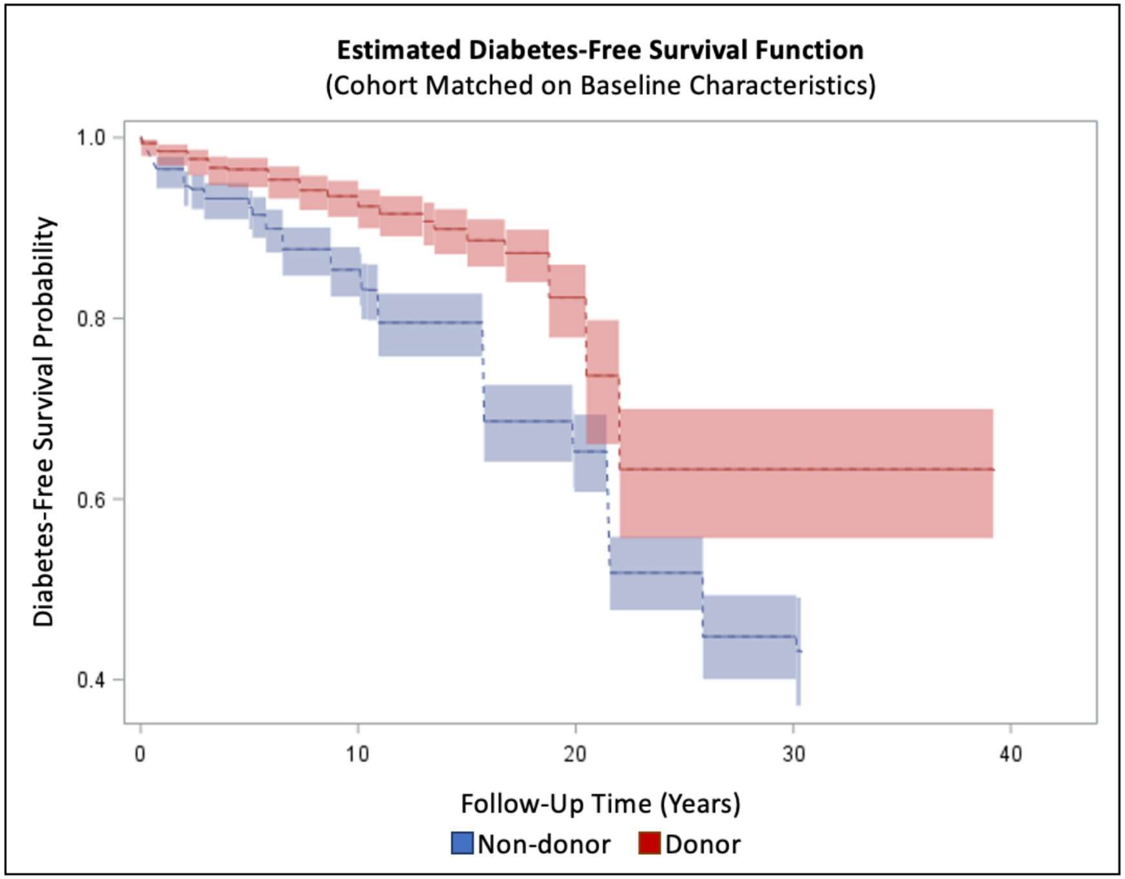

Figure S4. Non-parametric estimated survival models for diabetes-free survival utilizing full follow-up among cohort matched on baseline characteristics only (log rank $p<.0001$). Shaded area designates 95% confidence limits
